# Anti-Yo Antibody Uptake and Interaction with Its Intracellular Target Antigen Causes Purkinje Cell Death in Rat Cerebellar Slice Cultures: A Possible Mechanism for Paraneoplastic Cerebellar Degeneration in Humans with Gynecological or Breast Cancers

**DOI:** 10.1371/journal.pone.0123446

**Published:** 2015-04-17

**Authors:** John E. Greenlee, Susan A. Clawson, Kenneth E. Hill, Blair Wood, Stacey L. Clardy, Ikuo Tsunoda, Noel G. Carlson

**Affiliations:** 1 Neurology Service, George E. Wahlen Veterans Affairs Health Care System, Salt Lake City, Utah, United States of America; 2 Department of Neurology, University of Utah School of Medicine, Salt Lake City, Utah, United States of America; 3 Department of Microbiology and Immunology Center for Molecular and Tumor Virology (CMTV), Louisiana State University Health Sciences Center, Shreveport, Louisiana, United States of America; 4 Geriatric Research, Education, and Care Center (GRECC), George E. Wahlen Veterans Affairs Health Care System, Salt Lake City, Utah, United States of America; 5 Research Service, George E. Wahlen Veterans Affairs Health Care System, Salt Lake City, Utah, United States of America; 6 Department of Neurobiology and Anatomy, University of Utah School of Medicine, Salt Lake City, Utah, United States of America; INSERMU1138, FRANCE

## Abstract

Anti-Yo antibodies are immunoglobulin G (IgG) autoantibodies reactive with a 62 kDa Purkinje cell cytoplasmic protein. These antibodies are closely associated with paraneoplastic cerebellar degeneration in the setting of gynecological and breast malignancies. We have previously demonstrated that incubation of rat cerebellar slice cultures with patient sera and cerebrospinal fluid containing anti-Yo antibodies resulted in Purkinje cell death. The present study addressed three fundamental questions regarding the role of anti-Yo antibodies in disease pathogenesis: 1) Whether the Purkinje cell cytotoxicity required binding of anti-Yo antibody to its intraneuronal 62 kDa target antigen; 2) whether Purkinje cell death might be initiated by antibody-dependent cellular cytotoxicity rather than intracellular antibody binding; and 3) whether Purkinje cell death might simply be a more general result of intracellular antibody accumulation, rather than of specific antibody-antigen interaction. In our study, incubation of rat cerebellar slice cultures with anti-Yo IgG resulted in intracellular antibody binding, and cell death. Infiltration of the Purkinje cell layer by cells of macrophage/microglia lineage was not observed until extensive cell death was already present. Adsorption of anti-Yo IgG with its 62 kDa target antigen abolished both antibody accumulation and cytotoxicity. Antibodies to other intracellular Purkinje cell proteins were also taken up by Purkinje cells and accumulated intracellularly; these included calbindin, calmodulin, PCP-2, and patient anti-Purkinje cell antibodies not reactive with the 62 kDa Yo antigen. However, intracellular accumulation of these antibodies did not affect Purkinje cell viability. The present study is the first to demonstrate that anti-Yo antibodies cause Purkinje cell death by binding to the intracellular 62 kDa Yo antigen. Anti-Yo antibody cytotoxicity did not involve other antibodies or factors present in patient serum and was not initiated by brain mononuclear cells. Purkinje cell death was not simply due to intraneuronal antibody accumulation.

## Introduction

Paraneoplastic cerebellar degeneration in the setting of gynecological or breast malignancies is characterized clinically by progressive, ultimately profound cerebellar ataxia. Brains of affected patients demonstrate extensive—at times global—loss of cerebellar Purkinje cells with variable loss of granule and basket cells [1]. Sera and cerebrospinal fluid (CSF) from affected patients frequently contain high titers of antibodies, termed “anti-Yo” or “anti-Purkinje cell antibodies” (PCA1)”. These antibodies produce immunohistochemical labeling of Purkinje cell cytoplasm and recognize two proteins in Western blots of Purkinje cell lysates: a minor 34 kDa which is not invariably detected, and a major 62 kDa protein [2]. cDNAs encoding both minor and major antigens have been cloned and termed CDR34 and CDR62 respectively [3,4]. Anti-Yo antibodies also label cells within the tumors of affected patients, suggesting that these autoantibodies represent an immune response which is directed primarily against the underlying neoplasm but is also cross-reactive with Purkinje cell antigens [5]. Comparison of CSF and serum antibody titers has demonstrated synthesis of anti-Yo antibodies within the central nervous system of affected patients [6].

Although anti-Yo antibodies have been repeatedly demonstrated to be present in sera and CSF of affected patients, the role of these antibodies in causing Purkinje cell death has been uncertain. The Purkinje cell antigens recognized by anti-Yo antibodies are intracellular and have not been detected on Purkinje cell surface membranes [7,8]. Because neurons have been considered to exclude immunoglobulin G (IgG), it has been believed that intracellular Yo antigens are sequestered from antibody, thereby making anti-Yo antibodies unlikely contributors to the pathogenesis of Purkinje cell injury [9,10].

In previous studies, we have demonstrated that viable Purkinje cells in rat cerebellar slice cultures, incorporated—and also cleared—normal IgG [11]. We have subsequently investigated the interaction of anti-Yo antibodies with Purkinje cells in rat cerebellar slice cultures. This tissue culture system avoids the restriction of antibody access to CSF and brain normally imposed *in vivo* by the blood-brain barrier [12], and provides a model to study the direct pathogenic role of antibody in the absence of sensitized immune cells, including T cells. Using this model system we have previously demonstrated that patient IgGs containing anti-Yo antibodies were also taken up by Purkinje cells [12]. In contrast to normal IgG, however, anti-Yo IgGs were not cleared but rather were retained after binding to intracellular Purkinje cell antigens and induced cell death [12]. We also demonstrated that anti-Yo uptake and binding could be demonstrated in real time in viable cells and that antibody uptake could be blocked with colchicine [12]. These data suggested that anti-Yo antibodies may play a direct role in the pathogenesis of cerebellar injury. The present study addressed three further questions of importance in determining the role of anti-Yo antibodies in disease pathogenesis: 1) whether anti-Yo cytotoxicity specifically involved antibody binding to the 62 kDa major Yo antigen or might be caused by other antibodies or factors present in IgG from anti-Yo positive patients; 2) whether Purkinje cell death might be initiated by monocytes (macrophages/microglia) present in brain rather than by intracellular antibody binding; and 3) whether Purkinje cell death might be a nonspecific result of intracellular antibody accumulation in general and could be produced by other antibodies binding to other intracellular Purkinje cell antigens. We found that adsorption of antibodies to the 62 kDa Yo antigen abolished intracellular antibody binding and Purkinje cell death, indicating a direct role for these antibodies. Cells of macrophage/microglial origin did not infiltrate the Purkinje cell layer until death was already extensive. Incubation of cultures with a spectrum of antibodies specific for other intracellular Purkinje cell proteins resulted in uptake and intracellular antibody binding but did not cause cell death, indicating that cytotoxicity was not simply the result of intracellular antibody accumulation.

## Materials and Methods

### Patient Sera

Anti-Yo positive sera used as positive controls in this study were from 4 patients developing paraneoplastic cerebellar degeneration in the setting of ovarian or breast malignancies. Presence of anti-Yo antibodies and absence of other paraneoplastic autoantibodies was confirmed in all patients by demonstrating immunohistological staining of Purkinje cells typical for anti-Yo antibody in frozen and fixed sections of human and rat cerebellum and by antibody labeling restricted to the 34 kDa and/or 62 kDa proteins characteristic of Yo antigens in western blots of Purkinje cell lysates [2]. Specificity of autoantibody response was also confirmed by demonstrating reactivity with recombinant Yo protein. All sera had been previously shown to produce Purkinje cell death in rat cerebellar slice cultures [12]. Negative human control sera were obtained from normal individuals and from individuals with nonparaneoplastic neurological disorders [12]. To compare the specificity of anti-Yo cytotoxicity with human sera reactive with other Purkinje cell cytoplasmic proteins, we used sera from three patients with ovarian malignancies whose sera contained high titers of anticerebellar antibodies not reactive with Yo antigens in Western blots or recombinant Yo proteins; all three patients had remained neurologically normal until their deaths from the underlying cancer [13–15]. Two of these sera, from individuals with ovarian adenocarcinoma (Patients 1 and 2), produced immunohistological labeling of Purkinje cells identical to that produced by anti-Yo antibodies but recognized proteins of 66 and 90 kDa (Patient 1) and of 58 and 70 kDa (Patient 2) in western blots of Purkinje cell lysates [13,15]. Sera from patient 3, an individual with a mixed mesodermal sarcoma, produced nuclear and cytoplasmic labeling of Purkinje cells, basket cells, and occasional granule cells; and labeled proteins of 66, 80, and 120 kDa in Western blots of Purkinje cell lysates [15] (Greenlee et al. unpublished data). Purified IgG from patient sera was prepared by protein G column chromatography as previously described [16].

### Antibodies to defined Purkinje cell proteins

Uptake and cytotoxicity of commercial antisera to three intracellular Purkinje cell proteins were compared with anti-Yo antibodies: rabbit anti-calbindin antibodies (PA1-931; Thermo, Pittsburgh, PA), mouse anti-calmodulin (MA3-917; Pierce Antibodies, Rockford, IL) and goat anti-PCP-2 (also known as L7), a protein which is highly expressed in cerebellar Purkinje cells and retinal bipolar neurons (SC-49074; Santa Cruz Biotechnology, Inc., Dallas, TX) [17–20]. All commercially obtained antibodies were extensively dialyzed to remove azide prior to use in cultures and were used at 5 μg/ml. to approximate antibody titers similar to titers of anti-Yo antibodies typically found in CSF of affected patients.

### Organotypic cerebellar cultures

Organotypic cerebellar cultures were prepared at 200 μm from 10–12 or 23–27 day old Sprague-Dawley rats (Charles River, Germantown, MD) as previously described, using the method of Rothstein et al [11,21]. Horse sera used in tissue culture media was heated to inactivate complement. Cultures were incubated at 37°C in a 5% CO_2_/95% humidified air environment with twice weekly changes of medium. Resultant cultures exhibited typical cerebellar morphology, with Purkinje cells clearly identifiable by morphology and by positive immunostaining of Purkinje cells in fixed cultures with both anti-Yo antibodies and antisera to the Purkinje cell marker, calbindin-28k (Millipore, Temecula, CA) [22,23].

### Studies of anti-Yo IgG depleted of antibodies to the 62 kDa major Yo antigen

CDR62 is a recombinant cDNA originally cloned from a lambda zap library using anti-Yo antibodies and is believed to encode the major Purkinje cell antigen recognized by anti-Yo antibodies [4]. The 1365 bp coding region for the major Yo antigen (homo sapiens cerebellar degeneration-related protein 2, 62kDa, accession number BC017503) was sub-cloned into the plasmid pReceiver-B01 that contains a T7 promoter and a multiple cloning site which allows fusion of six histidine residues (His tag which facilitates binding to a nickel column) to the N-terminus of the protein. The resulting plasmid (EX-Z1407-B01) was constructed by GeneCopoeia (Rockville, MD) and transformed into the *E*. *Coli* strain BL21 (DE3)/pLysS. Post-induction cell pellets were frozen in buffer (NTB- Clontech) containing protease inhibitors, and the cell lysate was adsorbed onto a nickel affinity column (Clontech column # 635657). To provide a sham control for non-specific binding, the same cell lysate was adsorbed onto a nickel column prepared with bacteria containing a control vector which lacked the His-tag Yo antigen. The anti-Yo patient serum (diluted at 1:100 in organotypic culture media) was passed over the His-tag Yo antigen column or the sham control nickel column. Eluates from each column were tested by slot blot assays (spotted with recombinant His-tag Yo antigen) and showed that immunoreactivity to the Yo protein was removed when passed over the His-tag Yo antigen nickel column but was unaffected by passing over the sham column (data not shown). Eluates from the column with bound Yo antigen, the sham column, and the native, untreated serum were incubated at final IgG concentrations of 7.5 μg/ml.

### Incubation of cultures with patient and commercial antisera

Cultures were incubated at with either commercially obtained antibodies specific for three intracellular Purkinje cell proteins (calbindin, calmodulin, or PCP-2), or with sera from the three neurologically normal ovarian cancer patients whose anti-Purkinje cell antibodies did not react with Yo antigen [12]. Positive controls were cultures incubated in parallel with sera from 4 patients with confirmed anti-Yo antibodies previously shown to cause Purkinje cell death in culture [12]. Negative controls consisted of sera or purified IgG from patients without malignancy or neurological disease. Patient sera were diluted to IgG concentrations of 7.5 μg/ml, approximating amounts of anti-Yo IgG found in CSF of most patients with paraneoplastic cerebellar degeneration [12]. Commercially obtained and control IgGs, including sham-adsorbed anti-Yo IgG, were adjusted to the same concentration. Cultures were harvested at 24 hour intervals from 24 to 144 hours. All studies, unless otherwise indicated, were performed in triplicate.

### Determination of cell death and apoptosis

Determination of cell death was by adding the cell viability stains, SYTOX green or SYTOX orange, to cultures 2 hours before harvesting as previously described [11,12]. SYTOX dyes are compounds which are excluded from living cells but which readily enter dead or dying cells to bind to intracellular nucleic acids [24]. We have previously shown intracellular entry of these dyes to be a sensitive marker of Purkinje cell death [11,12]. Although we have previously demonstrated that anti-Yo antibody-mediated Purkinje cell death did not involve apoptosis [12], it was not known whether the other antibodies studied might produce apoptotic cell death. To identify cells undergoing apoptosis in cultures incubated with these antisera, cultures were incubated for the last 4 hours prior to harvesting with a 1:5 dilution of the pan-caspase substrate FLICA (carboxyfluorescein-labeled fluoromethyl ketone peptide substrate of caspases; Immunochemistry Technologies, LLC, Bloomingdale, MN) as previously described [12].

### Immunofluorescence methods

Cultures were fixed in 2% paraformaldehyde and permeabilized with 0.2% Triton X-100. To detect internalized IgG, cultures were incubated overnight with 1:800 dilutions of Cy5-conjugated donkey anti-human IgG (Jackson ImmunoResearch, West Grove, PA). Confirmation of intracellular antibody binding was obtained by examining contiguous serial confocal images of Purkinje cells within cerebellar slice cultures incubated with anti-Yo antibody for 24 and 48 hours. To identify microglia and macrophages, fixed and permeabilized cultures were incubated overnight with 1:200 dilutions of mouse anti-rat CD11b antibody (MCA 275R AbD Serotec, Raleigh, NC) and labeled with Cy3-conjugated donkey anti-mouse IgG (Alexafluor 494, Jackson Laboratories, Bar Harbor, ME).

### Quantification of Purkinje Cell Death

Cultures were incubated with anti-Yo sera, sham-adsorbed anti-Yo IgG, commercially obtained antisera reactive with other intracellular Purkinje cell proteins (calbindin, calmodulin, PCP-2/L7), sera from neurologically normal cancer patients with anti-Purkinje cell antibodies not reactive with Yo antigens (Patients 1–3), or normal human IgG. SYTOX green (25 nM) was added 2 hours prior to fixation and immunofluorescence confocal microscopy. The amount of cell death was quantified by an observer unaware of treatment of the cultures and consisted of counting of the number of cells labeled by Cy5 conjugated anti-human IgG which contained or lacked SYTOX green. Live cells were recorded as containing Cy5 IgG and lacking SYTOX green. Dead cells were scored as cells co-labeled for IgG and SYTOX green. Approximately 40–90 cells were counted for each field and the average percentage of cell death was obtained from a minimum of eight fields captured at 40X magnification for each time point [11]. Statistical significance between groups was determined by non-parametric Mann-Whitney *U*-statistical analysis using GraphPad Instat statistical software (GraphPad Software, Inc., LaJolla, CA).

### Confocal microscopy

To acquire confocal images, we employed a Nikon Eclipse E800 upright microscope (Nikon Biosciences, Melville, NY) and the Personal Confocal Microscopy PCM-2000, utilizing Argon-ion and green and red HeNe lasers as previously described [11,12]. Simple Personal Confocal Image software program (Compix, Cranberry Township, PA) was used to acquire digital images and image analysis. A red HeNe laser with a 633 nm excitation filter and 675 LP filter was used to visualize Cy5. The Argon-ion laser with a 514 nm excitation filter was used with a 510 LP filter to image SYTOX green. A green HeNe laser with a 543 nm excitation filter and a 565 LP filter was used to visualize SYTOX orange. The argon laser with a 488 nm excitation filter and a 510 LP filter was used to visualize FLICA. All filters were matched to the peak emission spectra of the fluorochromes employed. General procedures utilized individual fluorochromes with X, Y, and Z scans of 14–20 focal planes. Identical focal plane settings for each fluorochrome were used for single visual field analysis to ensure that each corresponding fluorochrome was imaged in the same focal plane. In all studies, stringent uniform experimental parameters and computer software setting were maintained for the respective image analyses. Because the vibratome preparation techniques used to prepare cerebellar slice cultures invariably resulted in death of neurons on the cut surfaces of culture slices, image analyses in all experiments were confined to the interior portions of the cultures.

### Ethics Statement

All human materials were obtained and studied under Institutional Review Board (IRB) guidelines (University of Utah and Veterans Affairs Salt Lake City Health Care System); and all procedures for acquisition and use of human materials were specifically approved by this IRB (IRB-approved protocol # 1919). Patient sera were obtained from two sources: 1) from individual patients providing written informed consent; or 2) as de-identified aliquots of patient sera obtained from ARUP laboratories. The consent form used and permission to use de-identified sera were both specifically approved by the institutional IRB as part of our protocol, and all consent forms were logged in and retained, with each patient being given a duplicate, signed copy of his or her consent form. Use of animals in this study was carried out in strict accordance with the recommendations in the Guide for the Care and Use of Laboratory Animals of the National Institutes of Health. All aspects of animal handling and care were conducted with approval of the George E. Wahlen Veterans Affairs Medical Center Salt Lake Animal Care and Use (IACUC) Committee, Permit # A12/19) and were conducted in an Association for Assessment and Accreditation of Laboratory Animal Care (AAALAC)-approved facility (The George E. Wahlen Veterans Affairs Salt Lake City Health Care System Veterinary Medical Unit). Euthanization of animals employed CO2 narcosis. All efforts were made to minimize suffering.

## Results

### Adsorption of antibodies to the 62 kDa major Yo antigen abolishes anti-Yo associated Purkinje cell cytotoxicity

**Fig 1:**
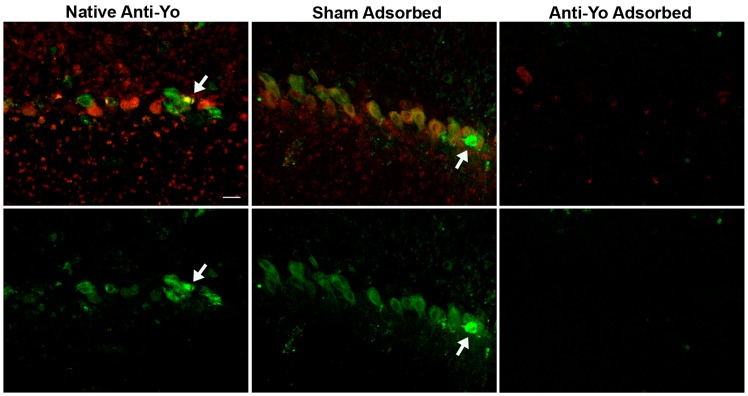
Adsorption of the 62 kDa major anti-Yo antibody abolishes intracellular accumulation of anti-Yo IgG within Purkinje cells and IgG-mediated cytotoxicity. In this figure the upper row shows merged images demonstrating both IgG accumulation (red) and entry of SYTOX dyes indicative of cell death (green; yellow indicates colocalization of IgG and SYTOX); the bottom row shows staining with SYTOX death markers only. Rat cerebellar slice cultures were incubated with either 1) patient serum containing anti-Yo antibodies (Native serum); 2) the same serum passed over a nickel column bound with lysates from bacteria containing a control vector which lacked the His-tag Yo antigen (Sham adsorbed); or 3) a nickel column with bound His-tagged 62 kDa Yo expression protein (Anti-Yo adsorbed). Cultures were evaluated at intervals through 72 hours for IgG accumulation within Purkinje cells and for Purkinje cell death. Incubation of cultures with untreated (Native) anti-Yo antibodies for 72 hours resulted in antibody accumulation within virtually all Purkinje cells (red) and in Purkinje cell death as indicated by intracellular penetration of SYTOX green (green; co-labeling appears yellow). Antibody uptake and killing were essentially identical in cultures incubated with native anti-Yo serum and sham adsorbed serum (see also Fig 2). In contrast, adsorption of sera with the 62 kDa Yo expression protein essentially abolished both antibody accumulation and cell death, indicating that Purkinje cell antibody accumulation and death are due specifically to interaction of anti-Yo antibodies with the 62 kDa cytoplasmic Purkinje cell protein. Scale bar = 20μ.

**Fig 2:**
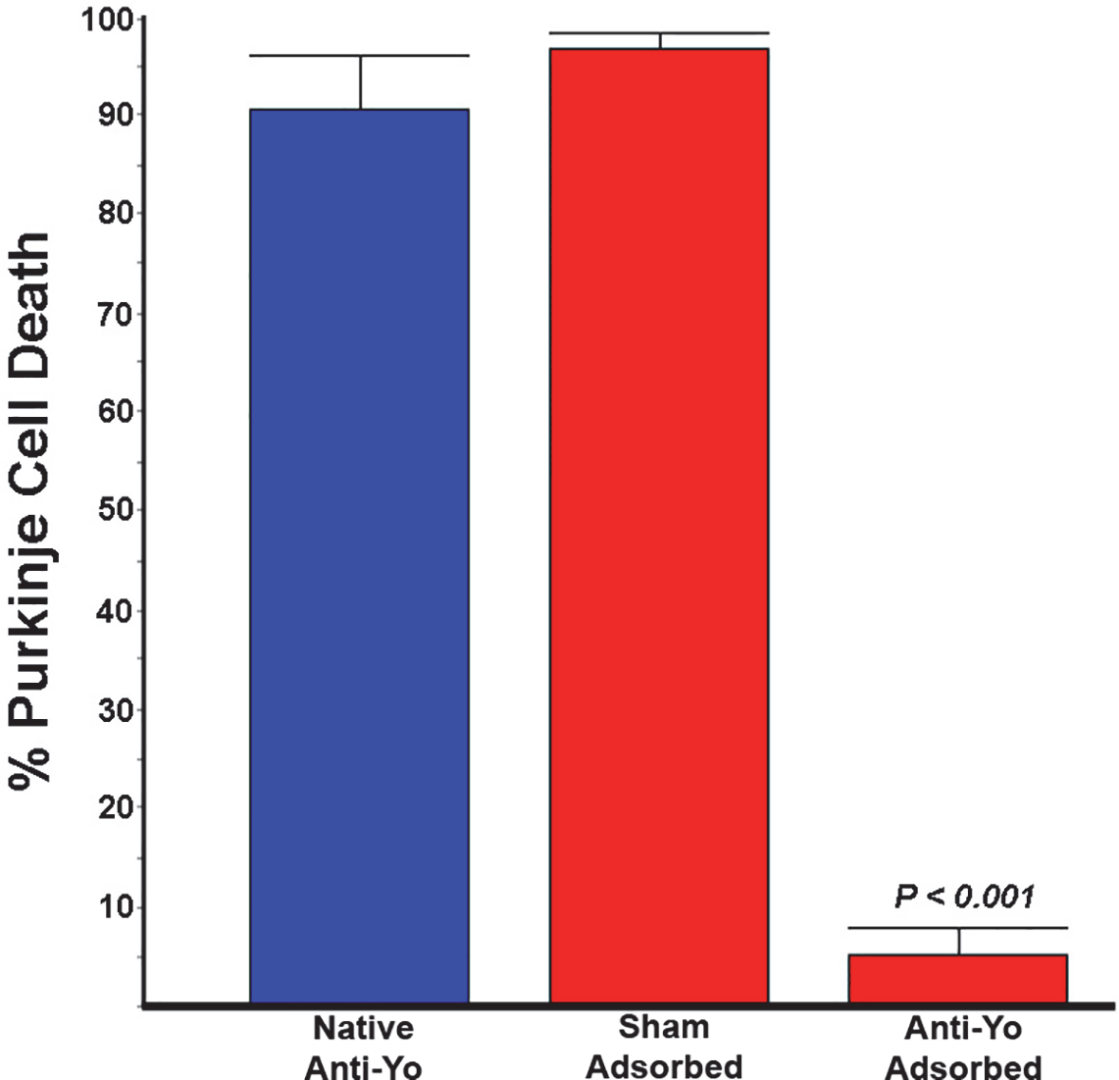
Quantitative analysis of Purkinje cell death in rat cerebellar slice cultures incubated with native anti-Yo serum (native serum), sham adsorbed serum, and serum adsorbed with the 62 kDa major Yo antigen. The per cent of Purkinje cells which contained IgG and stained with SYTOX green was counted from eight fields similar to that seen in Fig 1. Adsorption with the 62 kDa Yo expression protein effectively abolished Purkinje cell death compared to untreated serum or sham-adsorbed serum circulated through a nickel column with bound vector lacking Yo antigen.

To determine whether Purkinje cell death following incubation with anti-Yo sera required immunoreactivity to the 62 kDa Yo antigen, we depleted antibodies to the 62 kDa protein from patient IgG containing high titers of anti-Yo antibody [12]. Recombinant CDR62 major Yo protein with a N-terminus His tag (to facilitate binding to a nickel column) was bound to a nickel column, and patient serum containing high titers of anti-Yo antibody was passed through the column to deplete antibody to the 62 kDa protein. As a control for nonspecific binding to the column, the same serum was also passed over a nickel column treated with *E*. *coli* containing vector lacking Yo antigen (sham control). Rat cerebellar slice cultures were then incubated for 72 hours with native anti-Yo IgG, sham-adsorbed IgG, and IgG adsorbed against the 62 kDa protein. Cultures were monitored for antibody uptake and quantified for Purkinje cell death. Internalization of IgG by Purkinje cells was confirmed using serial confocal images through individual cells (S1 Movie). Cultures incubated with native anti-Yo IgG showed both extensive antibody uptake by Purkinje cells and Purkinje cell death, as did sham-adsorbed IgG in which antibodies to the 62 kDa protein were still present (Figs 1 and 2). In contrast, antibody uptake and Purkinje cell death were abolished following adsorption with the 62 kDa Yo antigen, indicating that antibody accumulation and Purkinje cell death were specifically associated with antibodies to the major Yo antigen (Figs 1 and 2).

### Microglia and macrophages accumulated near with Purkinje cells only after cell death was evident

**Fig 3:**
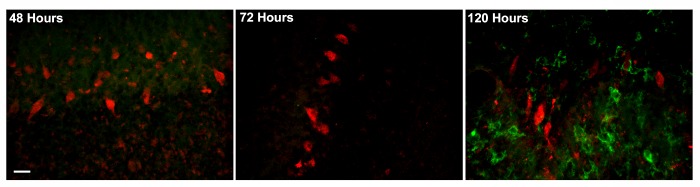
Macrophage/microglial cells infiltrate the Purkinje cell layer after cell death has already begun. The Purkinje cell layer is shown at three points in time. Cells of macrophage/monocyte lineage were not detected within the Purkinje cell layer at 48 hours, or at 72 hours, when cell death was already apparent. Macrophage/mononuclear infiltration did not begin until 96 hours (data not shown) and was more extensive at 120 hours. These data indicate that initiation of Purkinje cell death did not involve antibody-mediated cellular cytotoxicity. Scale bar = 20μ.

To determine whether Purkinje cell death might be due to interaction with brain mononuclear cells, cultures were incubated with anti-Yo antiserum, harvested at intervals between 48 and 120 hours, and co-labeled postfixation with anti-CD11b antibodies to detect macrophages/microglia. CD11b-positive cells were absent from the Purkinje cell layer at 48 hours and were still absent at 72 hours, when cell death was already widespread (Fig 3). Macrophage/microglial infiltrates became increasingly numerous as cell death became more extensive (Fig 3). These data indicate that Purkinje cell death preceded mononuclear cell infiltration, making it unlikely that brain mononuclear cells initiated Purkinje cell injury.

### Lack of Purkinje cell death after incubation with antibodies to other intracellular Purkinje cell proteins despite antibody uptake and binding

The anti-Yo antibody depletion studies described above demonstrated that Purkinje cell death was specifically dependent on antibodies reactive with the 62 kDa major anti-Yo antigen. However, these studies did not address the question as to whether cell death might be the result of intracellular antibody accumulation in general and could occur as a more general consequence of antibody binding to other Purkinje cell antigens. To address this question, rat cerebellar slice cultures were incubated with commercial antibodies reactive against three intracellular Purkinje cell proteins, calbindin, calmodulin, and PCP-2 as well as with sera from three ovarian cancer patients exhibiting serum anti-Purkinje cell cytoplasmic antibodies not reactive with Yo antigen (Patients 1, 2, and 3). Cultures were then followed for antibody accumulation within Purkinje cells and evidence of Purkinje cell death.

**Fig 4:**
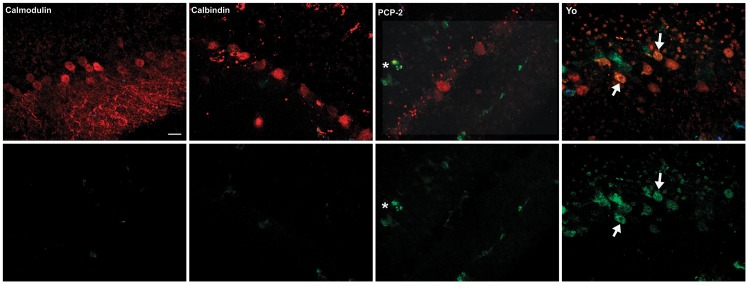
Comparison of uptake and cytotoxicity of anti-Yo antibody versus three other antibodies specific for the intracellular Purkinje cell proteins: calbindin, calmodulin, and PCP-2. The top row of panels demonstrates uptake and accumulation of IgG (red) within Purkinje cells after 96 hours in cultures incubated with anti-calmodulin, anti-calbindin, anti PCP-2, or anti-Yo IgGs. Entry of SYTOX green into Purkinje cells containing IgG, indicative of cell membrane injury and death (yellow), was seen in only in cultures incubated with anti-Yo IgGs (examples shown by arrows). The lower panels show only SYTOX green staining of Purkinje cells in cultures incubated with the antibodies indicated. In the culture incubated with anti-PCP-2. SYTOX staining indicative of cell death is seen in a single cell outside the Purkinje cell layer (asterisk) but not in Purkinje cells. Cultures were followed through 144 hours. There was progression of cell death in cultures incubated with anti-Yo antibodies. In contrast, cultures incubated calbindin, calmodulin, and PCP-2 did not exhibit Purkinje cell death above background levels seen in controls incubated with normal human IgG (data not shown). Scale bar = 20μ.

**Fig 5:**
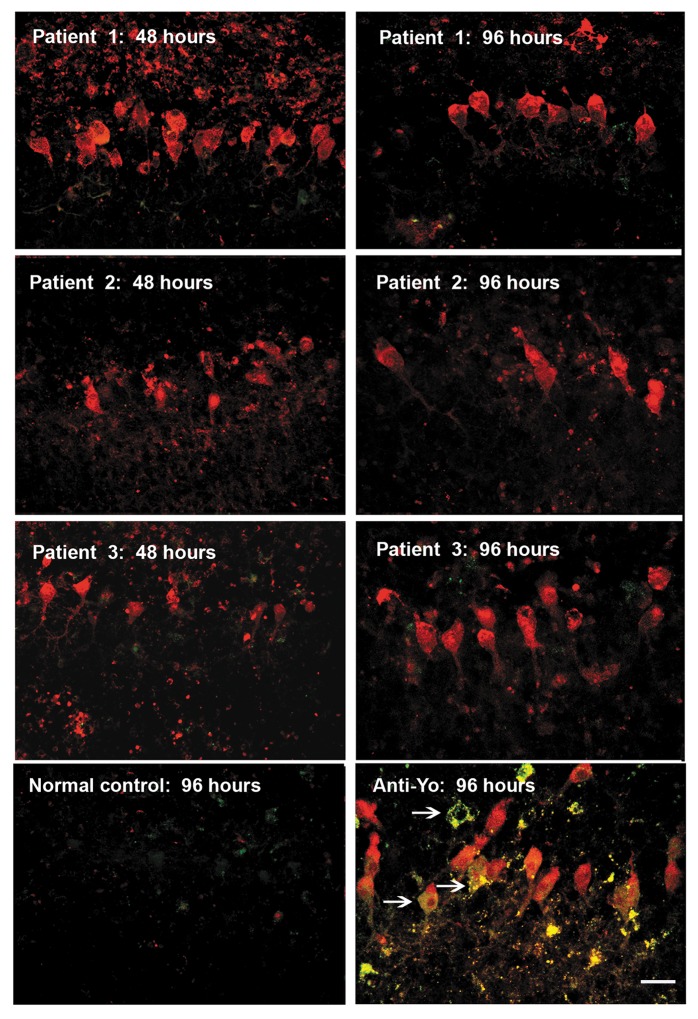
Comparison of uptake and cytotoxicity of anti-Yo antibody versus anti-Purkinje cell cytoplasmic antibodies obtained from three neurologically normal ovarian carcinoma patients whose sera did not react with Yo antigens. Uptake and accumulation of IgG (red) is seen with anti-Yo IgG and with IgGs from all three neurologically normal patients. Entry of SYTOX green into Purkinje cells containing IgG (yellow), indicative of cell membrane injury and death, is seen only in cultures incubated with anti-Yo IgGs for 96 hours (examples shown by arrows) but not with IgGs from neurologically normal Patients 1, 2, or 3. Uptake of normal IgG is not seen at confocal gain settings used for optimal visualization of anti-Yo antibody accumulation (13). Images shown are representative of three experiments. By the end of incubation (140 hours) staining for SYTOX green, indicative of necrotic cell death, was observed in less than 10% of cells incubated with sera from Patients 1, 2, and 3, an amount of background cell death staining similar to that seen in cultures incubated with control sera (data not shown). Parallel cultures incubated with anti-Yo sera or sera from Patients 1–3 sera remained negative by FLICA staining as compared to controls, indicating that with exposure to these sera did not result in apoptotic cell death (data not shown). Scale bar = 20μ.

**Fig 6:**
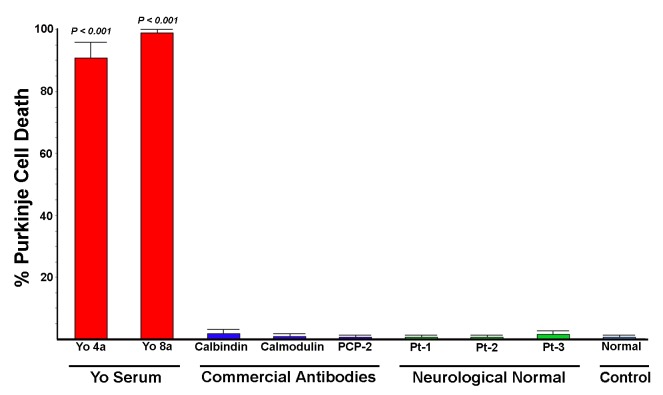
Comparison of Purkinje cell cytotoxicity produced by anti-Yo antibodies with cytotoxicity produced by commercial and patient antibodies reactive with other Purkinje cell cytoplasmic proteins. Rat cerebellar slice cultures were incubated for 72 hours with either 1) sera from patients with anti-Yo antibodies; 2) commercial antibodies to calbindin, calmodulin, or PCP-2/L7; 3) anti-Purkinje cells antibodies from the three neurologically normal patients studied, whose sera labeled Purkinje cell cytoplasm but did not react with Yo antigens; or 4) normal human IgG. Cultures were quantified for cell death as indicated by uptake of SYTOX dyes. Extensive Purkinje cell death was seen in cultures incubated with all 4 anti-Yo sera (data for 2 anti-Yo sera not shown). In contrast, cell death was not observed in cultures incubated with any of the three commercially obtained antibodies reactive with intracellular anti-Purkinje cell proteins nor with sera from Patients 1–3, despite extensive Purkinje cell uptake. Cultures incubated with normal human IgG exhibited only faint antibody accumulation by Purkinje cells and no detectable Purkinje cell death. Statistical significance between groups was determined by non-parametric Mann-Whitney ANOVA. Death in cultures incubated with anti-Yo antisera was statistically significantly greater than that seen in cultures incubated with commercial antisera, sera from patients 1–3, or normal human IgG.

**Fig 7:**
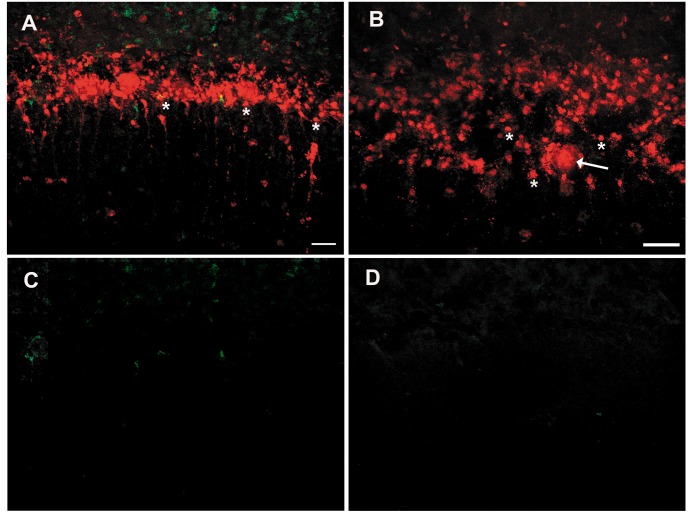
Rat cerebellar slice culture incubated with serum from Patient 3, a neurologically normal individual with mixed mesodermal ovarian sarcoma. **Panels A and B** demonstrate. extensive accumulation of IgG not only within Purkinje cells (arrow) but also in other neurons within and near the Purkinje cell layer (asterisks). **Panels C and D**: Purkinje and other neurons exhibiting Cy5 labeling for human IgG do not contain SYTOX green, indicating that IgG uptake had occurred in viable cells. Scale bars = 20μ.

As previously described, anti-Yo IgG was initially detected in Purkinje cell processes within 4 to 8 hours, then in Purkinje cell cytoplasm by 24 hours, and in Purkinje cell nuclei by 48 hours [12]. Uptake of IgG specific for calbindin, calmodulin, PCP-2, and IgG from patients 1, 2, or 3 was observed over this same time interval. At early time points (48 hours or earlier), Purkinje cells and other neurons containing IgG excluded SYTOX green, confirming cell viability at the time of antibody uptake. Although normal IgG is readily cleared from Purkinje cells, none of the intracellularly bound commercial or patient IgGs were cleared from Purkinje cells after removing unbound antibody from the media as is typically seen with normal human IgG [11]. By 48 hours, cultures incubated with anti-Yo antisera began to exhibit abnormal Purkinje cell morphology and by 72–96 hours, multiple Purkinje cells showed intracellular staining with SYTOX green, indicating cell membrane disruption and death (Figs 4, 5, and 6). Increasing numbers of Purkinje cells containing anti-Yo IgG and SYTOX green incorporation were observed at subsequent time intervals (data not shown) [12]. In contrast, although intracellular binding and accumulation of antibodies to calbindin, calmodulin, PCP-2, or from patients 1, 2, or 3 in cultures could be readily detected, incubation of cultures with these antibodies for periods of up to 144 hours did not result in detectable death of Purkinje cells in excess of background levels present in controls incubated with normal IgG (Figs 4, 5, and 6; data at 144 hours not shown). In cultures incubated with sera from patients 1 and 2 (with ovarian carcinomas), antibody uptake and accumulation were also observed almost exclusively in Purkinje cells. In cultures incubated with sera from patient 3 (with an ovarian mixed mesodermal sarcoma), IgG was detected not only in Purkinje cells but also in numerous small neurons within and near the Purkinje cell layer (Fig 7).

### Assay of cultures for neuronal apoptosis

In our prior studies using both TUNEL methods and FLICA staining, we demonstrated that Purkinje cell death following incubation with anti-Yo antibodies did not involve apoptosis [12]. To exclude the possibility that the commercially obtained antisera or the sera from neurologically normal patients might, unlike anti-Yo antibody, induce apoptotic cell death without SYTOX staining, cultures were incubated with each antiserum for 72 hours and were analyzed for the induction of apoptosis using FLICA staining as previously described [12]. FLICA fluorescence in cultures incubated with anti-calbindin, anti-calmodulin, or anti-PCP-2 antisera or with sera from Patients 1, 2, or 3 was only present at background levels, indistinguishable from that seen with control sera and from cultures incubated without antibodies (data not shown).

## Discussion

We have previously demonstrated that normal IgG was readily taken up by viable Purkinje cells and was also cleared from these cells without producing cell death [11]. In contrast, anti-Yo antibody was taken up by Purkinje cells, bound to intracellular proteins within these cells, and caused progressive cell death over time [12]. An important question in assessing the role of anti-Yo antibody in inducing Purkinje cell death was whether this cytotoxic effect was specific for the interaction of anti-Yo antibody with its major target antigen. The present study documents that depleting anti-Yo-specific IgG by adsorption of antibodies directed against the 62 kDa major Yo antigen virtually abolished Purkinje cell death. These data demonstrated that antibody binding to the 62 kDa cytoplasmic Purkinje cell protein was required for cell death, and that Purkinje cell killing by anti-Yo sera was not due to the presence of antibodies to other Purkinje cell antigens or to other potentially cytotoxic substances present in serum. Our study represents the first evidence that antibody interaction with the 62 kDa Yo antigen has a direct role in the pathogenesis of Purkinje cell death in paraneoplastic cerebellar degeneration and that Purkinje cell killing in this model did not require the presence of immune cells including sensitized T lymphocytes.

A second question in our study was whether death of Purkinje cells containing anti-Yo antibodies might be caused by interaction with cells of macrophage/microglia lineage. In our studies, infiltration of the Purkinje cell layer by macrophages/microglia did not occur until after cell death was already widespread, indicating that Purkinje cell death was not initiated by monocytes present in brain.

An additional question was whether Purkinje cell death in cultures incubated with anti-Yo IgG was unique to this antibody-antigen interaction or whether Purkinje cell death could result more generally from intracellular antibody uptake and accumulation and could be seen after incubation of cultures with antibodies to other intracellular Purkinje cell proteins. We found that multiple other antibodies reactive with intracellular Purkinje cell antigens were also taken up by Purkinje cells, but that intracellular binding and accumulation of these antibodies did not affect Purkinje cell viability. Our data thus indicate that Purkinje cell death following incubation with anti-Yo IgG was not simply due to intracellular antibody accumulation but was rather the result of specific antibody interaction with the 62 kDa Yo antigen.

The mechanism by which anti-Yo antibodies cause Purkinje cell death is not known. Hida et al., observing that Yo antigens were localized intracellularly on membrane-bound and free ribosomes, suggested that Purkinje cell death might be caused by interference with protein synthesis [7]. Although the 62 kDa Yo protein contains leucine zipper and zinc finger motifs commonly found in transcription factors and regulation of gene expression, the actual functions of Yo protein have not been elucidated by *in vivo* analysis of knockout animals or by other methods, nor has its intracellular localization been individually determined by electron microscopy [4]. Our studies, however, suggest that the 62 kDa antigen may serve an essential role in Purkinje cell survival, and that antibody binding to the antigen results in cell death.

In our prior studies employing normal IgG, we were able to detect IgG uptake only in Purkinje cells [11]. However, incubation of cultures with serum from Patient 3, with a mixed mesodermal sarcoma, resulted in intracellular binding of IgG in viable neurons other than Purkinje cells; this serum had been previously shown to label basket cells and other neuronal populations in fixed, permeabilized sections [15]. In subsequent studies, we have found that both anti-Hu and anti-Ri antibodies, which react with antigens present in essentially all neurons, are taken up by neurons throughout the CNS [25]. These observations, together with the present study, suggest that the ability to incorporate IgG may not be restricted to Purkinje cells but may be a property of CNS neurons in general. It is of note that Congdon et al. have also recently documented that neurons in transgenic mice expressing neuronal Tau protein can take up anti-Tau antibodies and that antibody trafficking within neurons provides a mechanism for Tau protein clearance [26]. Neuronal incorporation of IgG might thus allow other antibodies reactive with intracellular neuronal antigens to produce neuronal dysfunction or death and could conceivably allow antibody modulation of neuronal infection by viruses or other obligate intracellular pathogens. More work is needed to define the role of neuronal trafficking of IgG in human CNS disease.

## Conclusions

We have previously demonstrated that anti-Yo antibodies, associated with human paraneoplastic cerebellar degeneration, were taken up by Purkinje cells in rat cerebellar slice cultures and that antibody accumulation was followed by cell death. The present study is the first to demonstrate that cytotoxicity of anti-Yo antibodies for Purkinje cells required intracellular antibody uptake and binding to the 62 kDa cytoplasmic Yo antigen. Purkinje cell death was not initiated by brain macrophages or microglia, and was not produced by intracellular binding of other antibodies reactive with other cytoplasmic Purkinje cell proteins. These data indicate a primary role for anti-Yo antibody in disease pathogenesis. Our data also suggests that uptake and trafficking of IgG may occur in CNS neurons in addition to Purkinje cells, possibly providing a previously unrecognized mechanism for by which antibodies might affect intraneuronal processes.

## Supporting Information

S1 Movie Cytoplasmic binding and accumulation of anti-Yo antibody within Purkinje cells: Movie of contiguous serial confocal images of Purkinje cells within cerebellar slice cultures incubated with anti-Yo antibody for 24 and 48 hours. Images were made at 60X and 100X magnification. Antibody accumulation is detected in cytoplasm of virtually all Purkinje cells. Imaged cells excluded SYTOX dyes (data not shown), indicating that the antibody uptake had occurred in living cells.
(MP4)
